# Covid-19 and Instagram: Digital Service Innovation in Top Restaurants

**DOI:** 10.1007/978-3-030-65785-7_45

**Published:** 2020-11-28

**Authors:** Aarni Tuomi, Iis Tussyadiah, Mark Ashton

**Affiliations:** 1 Department for Informatics, Technical University of Munich, Garching bei München, Bayern Germany; 2 Smart Tourism Education Platform (STEP) College of Hotel and Tourism Management, Kyung Hee University, Seoul, Korea (Republic of); 3 Department of Tourism and Service Management, MODUL University Vienna, Vienna, Wien Austria; 4 Haaga-Helia University of Applied Sciences, Pajuniityntie 11, 00320 Helsinki, Finland; 5 University of Surrey, Stag Hill, Guildford, GU2 7XH UK

**Keywords:** Covid-19, Instagram, Digital service innovation, Restaurants

## Abstract

Governments across the world have imposed strict rules on social distancing to curb the spread of Covid-19. In particular, restaurants have been impacted by government-mandated lockdowns. This study adopts a mixed methods approach to explore how Finnish high-profile restaurants used Instagram as a means for service innovation and diffusion during nine weeks of government-mandated lockdown. Comparatively analysing 1,119 Instagram posts across two time-stamps (2019 and 2020) and across 45 restaurants, as well as conducting five semi-structured interviews with restaurant managers, it is found that while the overall number of Instagram posts and likes on posts stayed relatively similar to the year prior, the number of comments increased significantly, suggesting a move towards a more didactic and dyadic form of Instagram communication. In addition, four digital service innovation strategies are identified: launching new service offerings and introducing new elements to existing service offerings, fostering social relationship with customers, exploring novel streams of revenue, and reinvigorating the brand’s image. Implications to service innovation theory and practice are discussed, along with suggestions for future research.

## Introduction

2020 has been a very difficult year for the hospitality industry. The advent of severe acute respiratory syndrome coronavirus 2, more commonly referred to by the illness the virus causes, Covid-19, has forced governments around the world to impose strict restrictions on individual rights, particularly around the freedom of movement [1]. As the virus spreads primarily through human-to-human transmission, experts have concluded that the best way to curb infection rates is to introduce measures for social distancing. Hospitality, an industry which relies heavily on people moving around and interacting with each other, has been hit particularly hard by the pandemic [2]. In many countries, restaurants have been told to close or at the very least reduce the amount of covers served to meet social distancing requirements. In addition to scaling down operations and mass-furloughing staff, some food service operators have taken the crisis as an opportunity to innovate with new business models and service offerings. Information communications technology, in particular, has been seen as a potential tool for producing and providing socially distant services [3]. While the role of technology in innovation has been widely researched [4,5], previous studies mostly focus on disruptive changes brought by technology-driven (business model) innovation (such as Airbnb), and how incumbent firms are affected by and have responded to these changes [6]. Further, literature contrasts technology-driven vs. Market-driven (technology-push vs. Demand-pull) innovations in terms of their competitive and disruptive effects across the industry [5,7]. Service innovations we are witnessing today from hospitality firms are driven by the drastic change in the business environment (i.e., restrictions on physical operations), hence might not fit the mould of technology-push or demand-pull innovation. Furthermore, these innovations represent emergent strategies rather than planned, often involving a pivot to new business models at speed. This presents conceptual and practical challenges to better understand how technology and digital media play a role in the development of innovation for firms’ survival and competitiveness in the hospitality sector.

This study adopts a mixed methods approach to explore digital service innovation in Finland’s top 50 restaurants amidst Covid-19. In particular, the study addresses the following research question: How did Finland’s top 50 restaurants leverage technology to innovate their service offering and business model amidst government-mandated lockdown measures? Drawing from the literature on service innovation and organisational change, this study provides insight on the agility of largely low-tech, small- and medium sized companies’ innovation efforts, and offers important lessons learned as the sector prepares for the ‘new normal’ post-Covid-19.

## Service Innovation and Organisational Change

As discussed by Trott (2012) [8] innovation is, at the most basic level, about change. Change can be big or small, and it can take on different forms. For example, Voss and Zomerdijk (2007) [9] argue that service innovation may consider different elements of service: the service environment, the service employee, the service delivery process, fellow customers, or the back-office functions. Snyder et al. (2016) [10] see that service innovation may also involve the transformation of entire service products or service processes, leading to either incremental or radical innovations. Helkkula, Kowalkowski and Tronvoll (2018) [11] characterise service innovations into four archetypes: output-based service innovations, process-based service innovations, experiential service innovations, and systemic service innovations. Witell et al. (2017) [12] conclude that service innovation is fundamentally about the combining and recombining of an organisation’s available resources to improve its practices in novel, unforeseen ways.

Organisations go through change because the environments within which they operate are in constant flux. Dobbs et al. (2015) [13] see that modern companies are forced to change because of four key drivers in particular: urbanisation, technology, an ageing population, and globalisation. Besides these general megatrends of the 21^st^ century, Taleb (2008) [14] argues that sometimes change is due to what he calls a ‘black swan’ event. These are events that are rare, random, unexpected, and as such, extremely difficult to predict or plan for [14]. The emergence of Covid-19 could be characterised as a ‘black swan’ event because of its devastating impact on the global food service sector. Given the rapid pace and gravity with which hospitality operators have had to adapt to the new operating environment, an upsurge in emergent service innovation might be expected.

To conceptualise the roles of technology in innovation, extant research has largely agreed on the definition of technology-driven and market-driven innovations [5]. Technology-driven (technology-push) innovations happen when R&D experimentation precedes market opportunities, thus the potential market and applications are usually unknown [15]. Market-driven (demand-pull) innovations, even when involving technology, often result from radical changes in the value propositions made to existing customers [5]. Regardless of which came first, it is vital to align technology with user needs (demand) for innovations to be successful. Studying technology-driven innovations amongst retailers in the food sector, Esbjerg et al. (2012) [16] found that when implementing new (food) technologies, managers were driven by benefits to customers, confirming the importance of technology–demand alignment. Consequently, when this alignment is a challenge, firms, especially startups, are forced to redefine their competitive advantage and pivot their business model [17]. Garćia-Gutiérrez and Martinez-Borreguero (2016) [18] suggested the innovation pivot framework to guide firms navigating great uncertainty associated with internal and external factors. The framework assists in fostering the creative process of generating promising applications for an innovation by interrogating the links among the innovative technology, the sources of sustainable competitive advantage, and the innovative business model. Covid-19 presents an external challenge to existing firms due to the extreme narrowing of market and reduction in scale that has not been conceptualised fully in the literature on innovation and business model pivot. To address this gap in extant research it is important to examine the interplay between technological innovation and new demand exploration underlying service innovation and business model pivots in the hospitality industry as spurred by the pandemic.

## Methods

This exploratory study adopted a sequential mixed methods approach, choosing as its sample 50 high-profile restaurants across Finland. “Finland’s 50 best restaurants” is an annual compilation of the country’s top food service operators [19]. The prestigious ranking is produced in consensus with hundreds of Finnish hospitality professionals, from chefs to sommeliers, and from food critics to researchers. The 50 best restaurants list was deemed an appropriate sample for this study as it includes a wide variety of food service organisations that differ in size, service concept, business model, and geographical location. All restaurants on the list are also independent, i.e. not part of a large chain or a clear central brand. This was assumed to make the organisations particularly agile in implementing change and innovation and thus well-suited for this study.

Data were collected between June-July 2020, using two methods: (1) web scraping and (2) semi-structured interviews. After a comprehensive desk research phase to analyse all available online channels (e.g. website, social media platforms), it was concluded that the chosen sample of restaurants was most active on one digital channel in particular: Instagram. Put together, the restaurants have over 150,000 followers on the platform, with each individual organisation having thousands of followers and posting content several times per week. Others (c.f. [20]) have also found Instagram a rich source of information for hospitality research.

To understand how the restaurants’ business model and service offering changed during government-mandated lockdown measures, the restaurants’ Instagram posts were manually scraped across nine weeks pre-lockdown and during the lockdown: 30 March - 31 May 2020 (lockdown, calendar weeks 14–22) and 1 April - 2 June 2019 (normal operation, calendar weeks 14–22). In total, 1,119 posts (including the number of posts, likes, and comments as well as post captions) were scraped for analysis (2020: *n* = 554, 2019: *n* = 565). Number of posts, likes, and comments were compared week-by-week between 2019 and 2020, and the qualitative data found in captions were analysed thematically, identifying recurring patterns of behaviour/themes [21,22]. The analysis moved between deduction and induction [23], and drew from a priori themes established in recent innovation literature [11]. Out of the 50 restaurants, four were not found on Instagram or any other social media platform and thus were excluded from the study. One restaurant was found to have posted nine times the overall average in 2019 and not at all in 2020 and was thus marked as a clear outlier and excluded from the study. The final list of included restaurants (*n* = 45) along with their follower counts is shown in Table 1.

**Table 1.** Restaurants included in the study.

**No**	**Handle**	**Followers**
1	@palacehelsinki	4700
2	@basbasofficial	7500
3	@inarihki	5000
4	@restaurantolo	6300
5	@restaurantgron	13300
6	@ravintolavinkkeli	1700
7	@savoyhelsinki	3300
8	@ravintolakaskis	5200
9	@orarestaurantfin	3100
10	@restaurant_demo	2700
11	@ravintolac	1500
12	@restaurantkuurna	2200
13	@alexanderplatshelsinki	1900
14	@restaurant_tapio	5100
15	@ravintolamuru	5200
16	@ravintolamami	1400
17	@restaurantultima	4400
18	@finnjavel_finnishhautecuisine	7100
19	@restaurantnolla	9300
20	@kalliowino	3100
21	@bistro_omat	1200
22	@ostroferia	1900
23	@ravintelihuber	1500
24	@ravintelibertha	1900
25	@ravintolanokka	5800
26	@ravintolakakolanruusu	2900
27	@sicapelle_restaurant	1400
28	@gastrocafekallio	1400
29	@kajoravintola	2000
30	@kultakitchen	700
31	@pastisravintola	2600
32	@hellostrangerhki	1700
33	@yesyesyeshelsinki	4900
34	@farang_helsinki	3400
35	@ravintolaragu	2600
36	@ravintolapenelope	2900
37	@localbistro_jns	1400
38	@restaurantnude	2000
39	@maannos	1700
40	@restaurantspis	1400
41	@restaurant_gaijin	2400
42	@restaurantplein	2100
43	@ravintolasmor	1500
44	@ravintolaludu	1400
45	@oldboybbq	1700

**Fig. 1.**
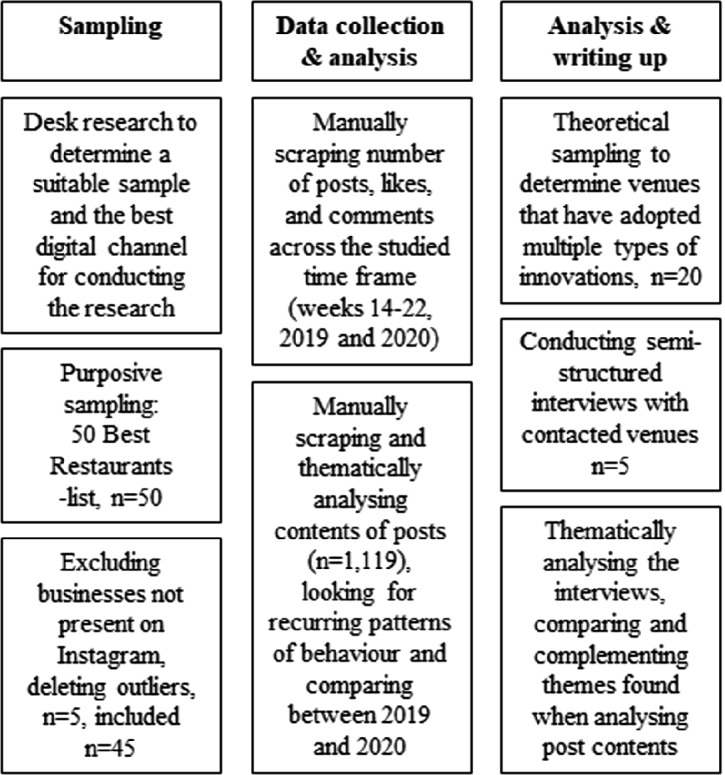
Data collection and analysis procedure.

To shed further light on the studied restaurants’ innovation strategies during the lockdown, theoretical sampling was conducted, whereby 20 of the most innovative restaurants were contacted to arrange a formal interview. In the end, representatives from five restaurants (three restaurant managers and two executive chefs/owners) took part in a semi-structured interview. The interviews lasted for 35 min on average and were conducted via phone and a video conferencing application. Questions focused on themes identified when analysing scraped posts, and dug deeper into the impacts of Covid-19 at a particular venue throughout the lockdown period. In particular, the researchers were interested in better understanding the challenges and successes of implementing innovation. The interviews were recorded, automatically transcribed, and as before analysed drawing on a priori themes established in recent service innovation literature [11]. Figure 1 provides a summary of the data collection and analysis procedures adopted herewith.

## Findings and Discussion

**Fig. 2.**
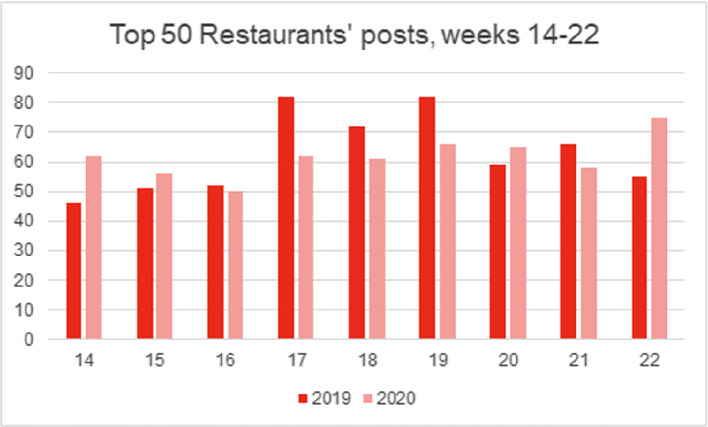
Total posts across the studied period (2019: *n* = 565, 2020: *n* = 554).

**Fig. 3.**
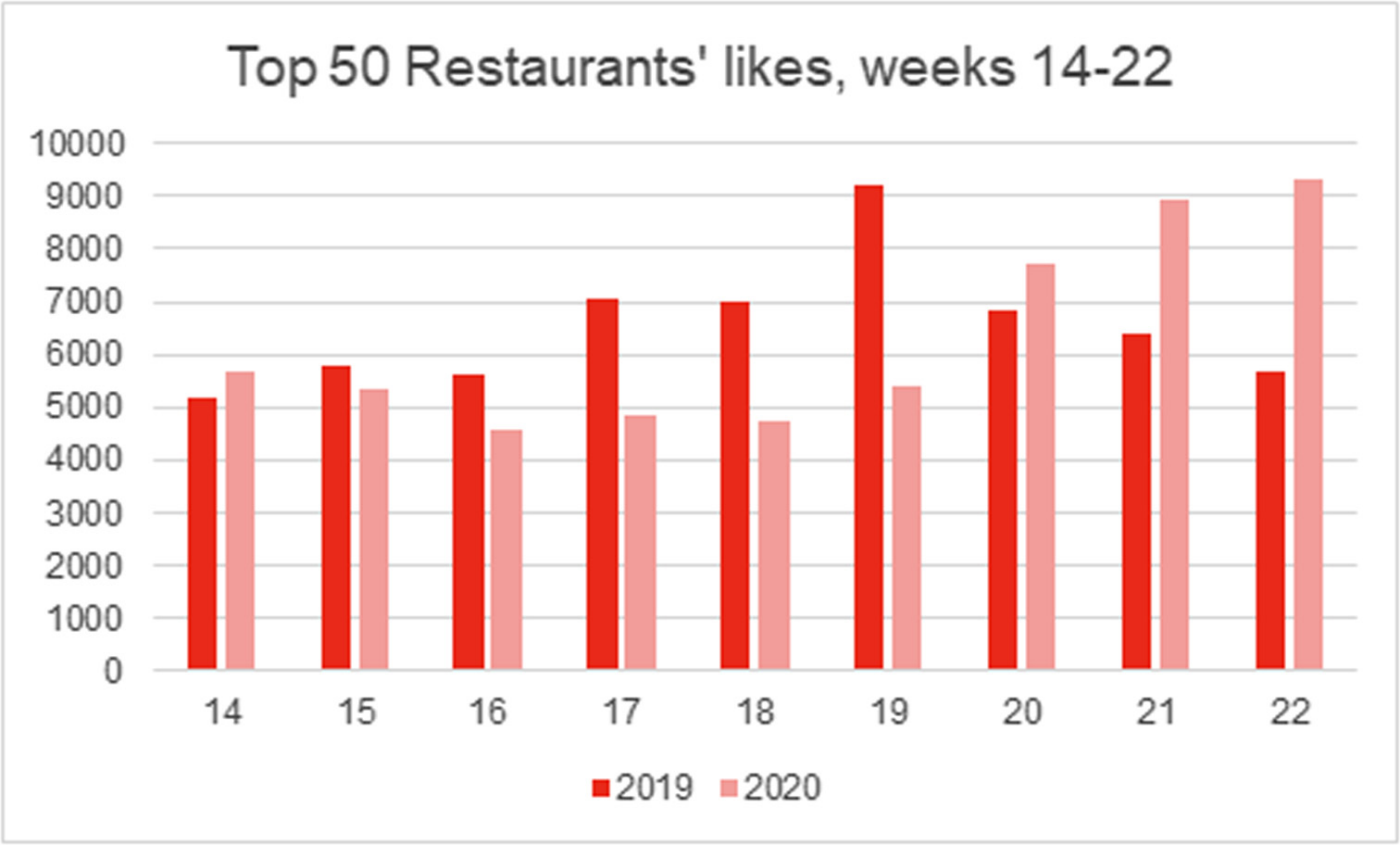
Total likes across the studied time period (2019: *n* = 58,829, 2020: *n* = 56,537).

As illustrated in Figs. 2 and 3, restaurants’ general post frequency and the overall number of likes per post did not significantly change between the lockdown and the year prior, although some variance was observed (i.e., some individual restaurants posted more during lockdown, while others posted less). However, the thematic content analysis highlighted a clear shift in the way in which Instagram was used as a customer-facing communication channel. While in 2019 posts were mainly promotional in nature, in 2020 posts were more dyadic, didactive, and often centred around the launch of a new service process or offering. As illustrated by one interview participant: *“Instagram quickly became our primary means of staying in touch with our guests. We would post about anything and everything, stuff we wouldn’t normally* […] *wouldn’t normally post”.*

**Fig. 4.**
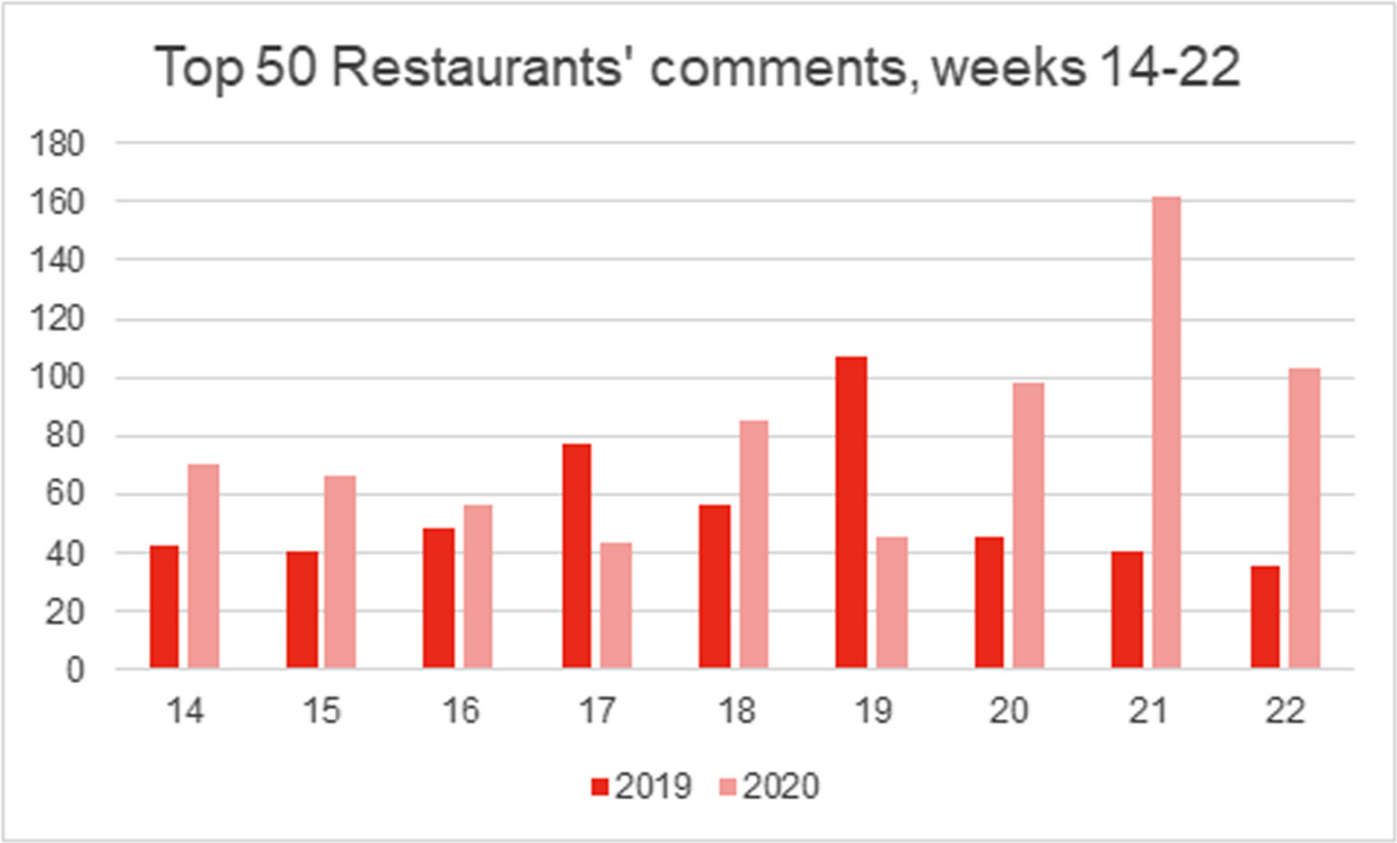
Total comments across the studied time period (2019: *n* = 497, 2020: *n* = 731).

The shift in Instagram usage is well illustrated by the peaks of post frequency, whereby in 2019 posts peaked during Finnish public holidays that fall within the studied time period (Easter on week 15/16, Labour Day on week 18, and Mother’s Day on week 19), while in 2020 post frequency was more constant with only slight peaks at the beginning and the end of the lockdown. The week-by-week number of comments per post provided further support for this, whereby a stark increase (32%) in comments was observed between 2019 and 2020 (Fig. 4). Posts towards the end of the lockdown (weeks 21–22) were commented particularly often, indicating a focus on re-establishing customer relationships and building trust.

Further noting the significance of Instagram as a two-way communication channel, one restaurant manager commented: *“Our Insta really blew up during the lockdown, we gained so many new followers, most organically. When the lockdown hit we basically lost all our reservations and catering orders overnight. We had no choice but to change our business and be damn quick about it. Insta effectively allowed us to stay in touch with our guests throughout the process.”*

In terms of the innovative service processes and offerings launched during lockdown, as observed from the thematic analysis of restaurants’ Instagram posts and the five semi-structured interviews with decision-makers, four distinct forms of digital service innovation were found: (1) launching completely new service offerings or introducing new value-adding elements to existing service offerings, (2) experimenting with new ways of fostering social relationships with customers, (3) exploring novel streams of revenue, and (4) focusing on revamping and reinvigorating the brand’s visual image or broader servicescape. These forms of service innovation resonate well with Helkkula, Kowalkowski and Tronvoll’s (2018) [11] four service innovation archetypes. As will be illustrated, in the first two types of digital service innovation identified herewith, Instagram forms a key part of the innovation itself and is thus used directly as an enabler of innovation, while in the latter two types of innovation Instagram is used primarily as a means of promoting the innovation to customers.

### Innovation Archetype 1 and 2: Output- and Process-Based Service Innovations

In terms of new service offerings, several high-end restaurants transformed their traditional tasting menus into a delivery-friendly format, either a ready-to-eat takeaway offering or a vacuum-packed meal kit. For example, Helsinki-based *Ora* transformed its Michelin-starred kitchen into a ‘sushi factory’, while *Demo* introduced a luxurious finish-at-home tasting menu complete with video instructions, wine pairings, and a specially curated Spotify-playlist to create the’right’ ambiance. Besides new offerings, restaurants also explored ways of bringing added value to the stay-at-home experience. Helsinki-based *Savoy* used the lockdown to educate their Instagram followers about the history of the restaurant, while Turku-based *C* took the opportunity to highlight the importance of working with local suppliers at a time when global supply chains are being disrupted. One of the most creative approaches came from *Finnjävel*, which decided to offer its customers a complimentary two-week access to an online course promoting and providing tools for taking care of mental health amidst the pandemic. In these cases, digital platforms (chiefly Instagram) play a role as enabler of innovation, allowing these restaurants to pivot their business model and generate new service offerings (e.g. reservation app for takeaway and delivery). Further, the availability of technology (e.g. a combination of Instagram videos and Spotify playlist) also allows businesses to afford implementation of emergent strategies to react quickly to the drastic change in the business environment. In other words, technology allows firms to quickly redefine their competitive advantage by exploring alternative use of their resources (i.e., capabilities, assets, knowledge) or pivoting the ways they reach customers and, consequently, operate.

### Innovation Archetype 3: Experiential Service Innovations

As highlighted by the shift to a more dyadic use of Instagram, restaurants fostered social connection through, for example, holding regular raffles or competitions. For example, Porvoo-based *Sicapelle* invited its followers to share pictures of their takeaway picnic setups, whereas in Turku Restaurant *Mami* held frequent raffles in collaboration with a local leafy green producer. In terms of more direct outreach, *Sikke*’s organised virtual champagne tasting events, while *Penélope* introduced virtual, home cooking-centred Instagram Live sessions and IGTV videos hosted by executive chef Hans Välimäki. Similar to the previous type of innovations, Instagram (and similar consumer-facing platforms) is used as a channel to enable the implementation of innovations as firms’ emergent strategies. Again, it signifies the role of technology as enabler of firms’ dynamic capability to stay highly flexible in the business world.

### Innovation Archetype 4: Systemic Service Innovations

Hospitality is a business with notoriously thin margins, and making ends meet even with new service offerings and innovative ways of staying in touch with customers is difficult. For this reason, the majority of the top 50 restaurants also experimented with additional revenue streams. From gift certificates to wholesale of branded products such as spice mix, beer, or hoodies, restaurateurs explored creative approaches to stay afloat. Several restaurants started a collaboration with a local supermarket to offer a branded ready-to-eat offering, while some turned to crowdfunding. Operators with multiple venues tended to focus their assets on one. Even though across the board many organisations were forced to furlough and lay off staff to cut costs, some came up with creative ways of utilising new-found time and manpower. Joensuu-based *Local Bistro* turned to volunteering and provided free meals for frontline healthcare staff, while in Helsinki Ultima took the opportunity to renovate premises and redesign customer journeys to facilitate a more low-touch post-lockdown service experience. As noted by one restaurant manager: *“The changes we implemented are here to stay. We simply cannot sustain our business at just half capacity once we’re allowed to take customers in again. We have no choice but to go with a more resilient hybrid model”.* The role of Instagram is less pronounced in these systemic service innovations. In fact, these emergent strategies are born from the lack of economic incentives in the business world, forcing businesses to realign their revenue model and mobilise their value network differently (e.g. supporting social causes) to remain sustainable. Whereas in other types of service innovation Instagram played a key part in enabling the innovation, in the case of systemic service innovations Instagram was used primarily for promotion.

## Conclusion, Limitations and Further Research

The coronavirus has managed to both devastate and develop the hospitality sector. On one hand, several restaurateurs have been forced to scale down operations, furlough and lay off staff, or even close. On another, the virus has hastened digitalisation efforts across entire industries, with food service operators that remain open having to come up with innovative new offerings and business models. This mixed methods study explored how Finland’s top restaurants reacted and adapted to a period of radical, exogenous ‘black swan’ event, specifically focusing on the roles of technology in these innovations. The innovations found in this study fit the categories specified in the literature [11]: output-based, process-based, experiential, and systemic process innovations. These innovations are the manifestation of emergent or discovery-driven strategies [5] largely enabled by the availability of technology and digital social media platforms. This suggests the vital role of technology in fostering dynamic capability of firms facing a drastic change in the business environment. As the lockdown period drastically reduces the scale of operations and the size of market (due to physical distancing), technology allows firms to explore alternative uses of their resources and identify different ways to secure their market foothold.

Despite offering important theoretical and practical insight on digital service innovation in the face of the unexpected, the study has limitations that need to be considered. First, only high-profile independent restaurants were analysed here. Extending the study to include operators with multiple centrally branded sites would have provided interesting insight into digital service innovation across large, less agile organisations. The study also focused solely on restaurants located in Finland. Replicating the study in other cultural contexts with different norms and conventions on service culture would produce a fuller picture of innovation efforts. Third, the study only focused on Instagram. Even though a set of interviews was conducted to gain a deeper account of the phenomenon studied, conducting content analysis on Instagram is inherently problematic. This is because of the ever-evolving nature of the platform, whereby there is no straightforward way of knowing whether old posts have been edited or deleted, when a particular post (old or new) has been liked or commented on, or how changes in the follower base (increase or decrease) might impact the overall number of likes or comments on a particular post. A more comprehensive look into different digital platforms used to generate and communicate service innovations in the hospitality sector will shed more light into the role of technology in service innovations.
